# Stem Rust Resistance and Resistance-Associated Genes in 64 Wheat Cultivars from Southern Huanghuai, China

**DOI:** 10.3390/plants13162286

**Published:** 2024-08-17

**Authors:** Yifan Wei, Xianxin Wu, Dongjun Liu, Huiyan Sun, Weifu Song, Tianya Li

**Affiliations:** 1 College of Plant Protection, Shenyang Agricultural University, Shenyang 110866, China; 2022240660@stu.syau.edu.cn (Y.W.); 2017101205@stu.syau.edu.cn (H.S.); 2 Institute of Agricultural Quality Standards and Testing Technology, Liaoning Academy of Agricultural Sciences, Shenyang 110161, China; 3 Institute of Crop Resources, Heilongjiang Academy of Agricultural Sciences, Harbin 150086, China

**Keywords:** wheat, *Puccinia graminis* f. sp. *tritici*, resistance gene, molecular detection

## Abstract

Wheat stem rust, caused by *Puccinia graminis* f. sp. *tritici* (*Pgt*), is a devastating fungal disease that affects wheat globally. The planting of resistant cultivars is the most cost-effective strategy for controlling this disease. The Huanghuai region, as a major wheat-growing area, plays a crucial role in the spread and prevalence of wheat stem rust in China. In this study, 64 wheat accessions from this region were tested at the adult stage against two major *Pgt* races, 34MKGQM and 21C3CTHQM. DNA markers associated with the known resistance genes *Sr31*, *Sr24*, *Sr25*, *Sr26*, and *Sr38* were measured to determine their presence in the tested accessions. In the 2023 field tests, 5 (7.8%) accessions were immune to 21C3CTHQM and 34MKGQM, while 35 (54.7%) and 39 (60.9%) were moderately resistant and resistant, respectively. The remaining 20 (30.7%) accessions were moderately susceptible and susceptible. In the 2024 tests, 12 (18.8%) and 14 (21.9%) entries were immune to both races; 29 (45.3%) and 30 (46.9%) were moderately resistant and resistant, respectively. Only two cultivars, Xinong 816 and Yimai 211, were immune in both years, and three entries showed some degrees of resistance in both years. Seven cultivars, including Zhongzhimai 23, Longxing 1, Yunong 937, Huaguan 301, Wanke 800, Shaanhe 285, and Yunong 612, showed increased susceptibility. DNA markers showed that 30 entries carried *Sr31*, while 6 entries carried *Sr38*. Genes *Sr24*, *Sr25*, and *Sr26*, which confer good resistance to the globally prevalent cultivars TKTTF and TTTRF, were absent from the set of tested entries. While this study surveyed the resistance levels of a cross-section of wheat from the southern part of the Huanghuai region and confirmed the presence of two known resistance genes, the basis of immunity or high levels of resistance in several lines remains obscure.

## 1. Introduction

Wheat is one of the world’s most important food crops and is widely cultivated almost around the world, with a range of cultivars possessing different characteristics [1]. Wheat cultivation and yield are crucial for maintaining national food security, promoting economic development, and improving living standards. Wheat stem rust caused by *Puccinia graminis* f. sp. *tritici* (*Pgt*) poses a significant threat to wheat production [2,3,4,5,6,7]. It is found in most regions of the world and causes severe damage to wheat crops [8]. It is estimated that 66% of global wheat-planting areas are susceptible to wheat stem rust, risking severe losses [9,10,11,12,13]. However, since the 1970s, there has been some control of the disease through the cultivation and promotion of resistant cultivars and the manipulation of resistance-associated genes [14,15,16,17]. Nevertheless, sudden outbreaks can occur, and thus continuous monitoring remains essential [18]. Major epidemics of wheat stem rust in China are associated with the emergence of new virulent races, the loss of resistance in key cultivars, and the widespread cultivation of susceptible lines [19]. Therefore, the cultivation and breeding of resistant cultivars are the most economical and effective measures for preventing and controlling wheat stem rust.

Resistance to stem rust in wheat is significantly associated with the *Sr31* gene. However, the *Pgt* race Ug99 has shown virulence against *Sr31* [20]. Ug99 collections in Kenya were designated as TTKS based on the North American nomenclature system and later as TTKSK after adding a fifth set of differentials [21]. The emergence of Ug99 underscores the threat posed by stem rust to global wheat production. Ug99 was recently found to have virulence not only against *Sr31* but also against several other resistance genes in wheat, including *Sr24*, *Sr38*, and *Sr36*, as a result of successive mutations [5,22]. In the 21st century, wheat stem rust, which had been absent from Europe for many years, reappeared. Between 2013 and 2014, TKTTF was detected in Germany, the United Kingdom, and Denmark. In 2016, thousands of hectares of durum wheat in Sicily, Italy, were affected by stem rust, the first significant outbreak of the disease in the region for 50 years [23]. In 2020, wheat stem rust was detected in various regions of Switzerland, with transcriptomic analysis revealing a close genetic relationship between this strain and TKTTF [24]. With the spread of Ug99 and its variants in African countries and the emergence of TKTTF and TTTTF in Europe, wheat stem rust has once again become a major concern [23,24,25].

The Huanghuai region of China is the country’s largest wheat-producing area and has played a significant role in the epidemiology of wheat stem rust in China. Understanding the resistance of local cultivars (lines) and the expression of their disease-resistance genes is crucial [26]. This study aimed to evaluate the resistance of 64 wheat cultivars collected from the southern part of the Huanghuai region, China, using two major *Pgt* races, 21C3CTHQM and 34MKGQM [25]. Additionally, the molecular markers associated with the *Sr* genes *Sr31*, *Sr24*, *Sr25*, *Sr26*, and *Sr38* were measured to assess the presence of these resistance genes. The research findings provide a reference for the prevention and control of wheat stem rust and for the breeding of resistant cultivars.

## 2. Results

### 2.1. Field Evaluation

Field assessments of resistance were conducted during 2023 and 2024, evaluating the infection rates (IRs) of 64 wheat accessions with two major *Pgt* races, 34MKGQM and 21C3CTHQM (Table 1). Resistance identification usually begins at the end of May, when environmental temperature and humidity (Supplemental Table S1) are in the optimal period for the occurrence and spread of wheat rust. The results of the identification can accurately reflect the resistance of the wheat accessions to the tested races of *Pgt*. The results indicated that in 2023, five accessions were immune to 21C3CTHQM and 34MKGQM; 35 and 39 accessions were resistant against each of the races, respectively; and 24 and 20 accessions, respectively, were classified as susceptible (Table 2). In 2024, 12 and 14 accessions were immune to races 21C3CTHQM and 34MKGQM, respectively, while 29 and 30 accessions displayed resistance, with 23 and 20 accessions were classified as susceptible, respectively. Both races exhibited strong virulence against the evaluated cultivars. Notably, only two cultivars, Xinong 816 and Yimai 211, retained immunity over the two years, while the Xuyan 6, Xinong 1522, and Daimai 519 cultivars showed consistently high resistance during this period. In contrast, seven cultivars (Zhongzhimai 23, Longxing 1, Yunong 937, Huaguan 301, Wanke 800, Shaanhe 285, and Yunong 612) showed significant susceptibility over the two years.

### 2.2. Molecular Markers of Stem Rust Resistance Genes in Cultivars (Lines)

**Figure 1:**
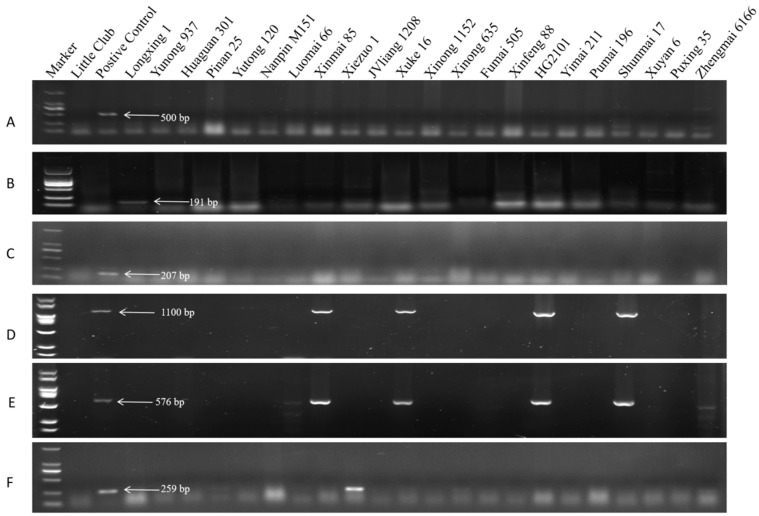
Amplification of *Sr24*, *Sr25*, *Sr26*, *Sr31*, and *Sr38* genes in 21 wheat cultivars. (**A**–**F**) Amplification of *Sr24*, *Sr25*, *Sr26*, *Sr31*, and *Sr38*, respectively, in 64 wheat cultivars. The positive control strains used were *Sr24* (Sr24#12), *Sr25* (Gb), *Sr26* (Sr26#43), *Sr31* (Iag95), *Sr31* (SCSS30.2_576_), and *Sr38* (VENTRUP-LN2).

The molecular markers associated with *Sr24* (Sr24 # 12), *Sr25* (Gb), *Sr26* (Sr26 # 43), *Sr31* (Iag95, SCSS30.2_576_), and *Sr38* (VENTRIUP-LN2) were measured in the 64 wheat accessions to determine the presence of these genes related to stem rust-resistance. The results revealed specific bands at 500, 191, and 207 bp, representing *Sr24*, *Sr25*, and *Sr26*, respectively, with 576 (SCSS30.2_576_) and 1100 (Iag95) bp corresponding to *Sr31*, and 259 bp indicating *Sr38*. None of the 64 wheat entries showed amplification of the bands linked to *Sr24*, *Sr25*, and *Sr26* (Figure 1A–C), while amplification of the band corresponding to *Sr31* was seen in 30 entries. Only six entries showed amplified bands representative of *Sr38*. In summary, none of the wheat entries were found to contain *Sr24*, *Sr25*, or *Sr26*, while 30 entries contain *Sr31*, and six contain *Sr38*.

## 3. Discussion

*Sr24*, which originates from *Thinopyrum ponticum*, is a primary resistance gene against stem rust in wheat cultivars grown in South Africa, Kenya, Australia, the United States, Europe, and the International Maize and Wheat Improvement Center (CIMMYT) in Mexico. The continuous mutation of Ug99 has led to the emergence of the new races TTKSK and TTKSP, which can overcome the resistance provided by *Sr24* and thus pose a significant threat to wheat production [27]. While some *Pgt* races exhibit virulence against *Sr24*, wheat cultivars carrying this gene still show good resistance to the new races TKTTF and TTRTF [28]. In this study, a specific 500 bp fragment was amplified using Sr24#12 primers in a positive control. However, this fragment was not detected in the 64 wheat cultivars (lines) tested, indicating that *Sr24* was not present in these cultivars [29,30,31,32]. Despite its common use in wheat resistance breeding in other parts of the world, this gene appears to be relatively rare among wheat cultivars in China. Previous molecular testing of 449 wheat cultivars from Henan, Shandong, Shaanxi, Gansu, Xinjiang, Heilongjiang, Inner Mongolia, and Yunnan revealed that only one cultivar, Kenong 1006, contained *Sr24*. Given the strong resistance of *Sr24* to Ug99, it is recommended that other resistance genes be aggregated and introduced into Chinese wheat cultivars during breeding efforts to enhance the genetic diversity of resistance traits in domestic cultivars.

The *Sr25* and *Sr26* genes are both known to provide excellent resistance to Ug99 and its variants, TKTTF and TTTRF, as well as to all races of *Pgt* found in China [31]. In recent years, these two genes have been used extensively in wheat resistance breeding throughout the world. However, *Sr25* is temperature-sensitive and provides reduced resistance in adult plants compared to seedlings, with raised temperatures significantly increasing the risk of susceptibility. *Sr26* is commonly employed in wheat breeding programs in Australia [32]. Based on previous molecular analyses and the findings of the present study, it appears that Chinese wheat cultivars generally lack both *Sr25* and *Sr26*. Therefore, to enhance resistance to the new Ug99 and its variants, it would be advisable to introduce these genes into domestic wheat via resistance breeding to expand the genetic pool of resistance traits in Chinese wheat cultivars.

The *Sr31* gene, located on the 1BL/1RS chromosome of wheat and transferred from rye “Petkus”, has been widely used in wheat breeding since its introduction in China during the 1950s. It is estimated that nearly 50% of wheat cultivars may contain this gene. Although *Sr31* has lost its resistance to the Ug99 race, it still maintains good resistance to the other races of *Pgt* found in China [29]. The present study found that 30 of the 64 tested wheat cultivars (lines) from this region contained this gene, indicating a significant presence of *Sr31* among Chinese cultivars. To date, no new races capable of overcoming *Sr31*-mediated resistance have been found in China [30,31,32]. Consequently, cultivars carrying this gene have played a vital role in maintaining the stable population structure of *Pgt* in the country. Despite the loss of resistance to Ug99, *Sr31* should still be judiciously employed in breeding programs for the effective prevention and control of stem rust recurrence in China.

The *Sr38* gene, originating from *Aegilops ventricosa*, is associated with the leaf rust-resistance gene *Lr37* and the stripe rust resistance gene *Yr17* [33]. It provides robust resistance against three types of rust in wheat and is therefore widely utilized in wheat rust-resistance breeding worldwide. Consequently, *Sr38* holds significant potential for future research on the prevention and control of wheat stem rust. Among the 64 wheat cultivars tested in this study, six were found to contain the *Sr38* gene. Given the crucial role of *Sr38* in preventing and controlling wheat stem rust in other parts of the world, as well as its marked resistance to wheat stem rust in China, these six cultivars could be used strategically with cultivars containing other resistance genes to enhance resistance in accordance with specific environmental conditions.

The present study evaluated the resistance of wheat cultivars from the southern part of the Huanghuai region to two *Pgt* races during 2023 and 2024. It was found that the resistance to the *Pgt* races 21C3CTHQM and 34MKGQM was poor. Of the 64 wheat cultivars tested, only Yimai 211 and Xinong 816 demonstrated immunity to both races during the 2023–2024 period. Among the tested materials, 20% of the accessions showed moderate susceptibility to susceptibility to the tested races, and some susceptible accessions had similar resistance levels to the tested race as the control variety LC, which had no resistance genes. The field severity and prevalence rates were as high as 100% (100S100). These plants began to dry up around 21–23 days after inoculation and had almost no yield at harvest. Molecular testing confirmed that 30 wheat cultivars (46.9%) contained the *Sr31* resistance gene, indicating the significant presence of this gene among Chinese cultivars. Additionally, six cultivars were observed to contain the *Sr38* gene. To date, no new races have been found to be capable of overcoming the resistance conferred by *Sr31* and *Sr38*. Therefore, cultivars harboring these genes have been instrumental in maintaining the stable population structure of *Pgt* in China. Furthermore, wheat cultivars such as Daimai 519, Xinong 1522, Yannong 5066, and Zhengmai 6166, which exhibit resistance to races 21C3CTHQM and 34MKGQM, may possess additional genes conferring resistance to these two races.

## 4. Materials and Methods

### 4.1. Plant Materials and Pgt Races

The 64 wheat cultivars were provided by Associate Researcher Cao Tingjie from the Henan Academy of Agricultural Sciences. The races 21C3CTHQM and 34MKGQM were isolated, identified, and preserved at the Plant Immunology Laboratory of Shenyang Agricultural University for this study (Table 3). The susceptible variety Little Club (LC) and the single-gene, positive control lines LcSr24Ag (*Sr24*), Agatha/9*LMPG (*Sr25*), Eagle (*Sr26*), Sr31/6*LMPG (*Sr31*), and Trident (*Sr38*) for molecular detection were also provided by the Plant Immunology Laboratory of Shenyang Agricultural University.

### 4.2. Filed Identification at the Adult Stage

The field test for identifying resistance in adult plants was set up during spring in the experimental wheat field of the Plant Protection College of Shenyang Agricultural University. All winter wheat underwent vernalization treatment prior to sowing. Specifically, the tested wheat cultivars were placed in a culture dish and covered with a double layer of filter paper to induce germination. After the seedlings reached a height of about 1 cm, they were refrigerated at 4 °C for approximately 20 days of low-temperature vernalization and were subsequently sown in a single row 1 m in length with a row spacing of 25 cm. The LC variety was planted on the periphery as an inducing and protective row.

During the bolting stage, the wheat was inoculated (inoculation dates: 28 May 2023, 27 May 2024) using the powder-spraying method, ensuring that the soil remained completely moist during inoculation, which was carried out at sunset. Before inoculation, the plants were wetted with an aqueous solution of 0.05% Tween 20. The stem rust spores were then mixed with talcum powder in a 1:30 ratio and sprayed onto the wheat plants. After inoculation, the plants were covered with plastic film and kept moist for 16 h.

The plants were inspected once every five days for a total of three inspections, after which the LC cultivar used to check susceptibility was fully diseased. The resistance and susceptibility levels were classified according to the standards for wheat stem rust response types (Table 4). The IR classification included I (immune), NI (nearly immune), R (very resistant), MR (moderately resistant), MS (moderately susceptible), and S (susceptible). Disease severity was assessed according to the percentage of the surveyed leaf area occupied by uredinia, using benchmarks of 0.37% of the leaf area occupied by uredinia to represent a severity of 1% and 37% of the leaf area covered by uredinia to indicate a severity of 100%. Disease severity was classified into 12 levels, namely, 1, 5, 10, 20, 30, 40, 50, 60, 70, 80, 90, and 100%. The climatic conditions are shown in Supplemental Table S1.

### 4.3. Molecular Markers of Resistance Genes

DNA was extracted from wheat using the CTAB method, and its quality was assessed by 1% agarose gel electrophoresis. Molecular markers closely linked to the resistance genes *Sr24*, *Sr25*, *Sr26*, *Sr31*, and *Sr38* were used to detect the presence of these genes in the 64 cultivars from the Huanghuai region. The primers used were synthesized by Sangon (https://www.sangon.com). The PCR amplification reaction system included 5 µL of Taq polymerase, 0.5 µL each of positive and negative primers, 1 µL of DNA, and ddH_2_O to make up 10 µL. The specific reaction conditions are detailed in Table 5.

## 5. Conclusions

This study evaluated the resistance of the adult plants of 64 wheat cultivars (lines) grown in the southern part of the Huanghuai region in China to two physiological races of *Pgt*. After the artificial inoculation of adult plants, it was found that 37 cultivars were resistant to both races 21C3CTHQM and 34MKGQM, while the remaining cultivars exhibited varying degrees of susceptibility. Molecular testing revealed that among these 37 resistant wheat cultivars, 32 contained either *Sr31* or *Sr38*, while five did not possess any of the tested resistance genes, suggesting that their resistance mechanisms require further investigation.

Notably, cultivars such as Nanpin Yongminmai 2, Juliang 1208, Yimai 211, Zhengmai 6166, Xuyan 6, Bainongchengzhu 21, Xinong 1522, Xinong 816, and Tianyike 18 demonstrated high resistance to both races, showing immunity to near immunity in field conditions. These cultivars could be utilized selectively in the breeding process. Due to the infrequent global occurrence of wheat stem rust, resistance to this disease has not been a primary breeding goal in many regions, leading to a shortage of cultivars with strong resistance. With the continuous emergence and spread of new races of *Pgt* worldwide, the disease is poised to become a significant concern once again, making the exploration of resistance resources critical.

This study analyzed the resistance and conducted molecular testing on 64 wheat cultivars in the southern region of Huanghuai, preliminarily clarifying their resistance to current stem rust races and the distribution and frequency of resistance genes. The findings provide a theoretical basis for wheat resistance breeding and the strategic deployment of resistance genes. However, given the limited number of tests currently available, many known stem rust resistance genes were not evaluated. Therefore, it is necessary to further enhance the detection of resistance genes in cultivars to facilitate rational planting and breeding.

## Figures and Tables

**Table 1:** Wheat accessions showing resistance and susceptibility at the adult plant stage to the two *Pgt* races during 2023 and 2024.

Cultivars (Lines)	Infection Responses ^a^				Results of Molecular Marker Tests ^b^					
	21C3CTHQM		34MKGQM							
	2023	2024	2023	2024	*Sr24*	*Sr25*	*Sr26*	*Sr31*		*Sr38*
								Iag95	SCSS30.2_576_	
Nanpinzhongmai 6321	0	0	50MR50	0	-	-	-	+	+	-
Zhongzhimai 22	50MR30	40MR10	50MR20	40R20	-	-	-	+	+	-
Zhongzhimai 23	100S100	100S100	70S70	100S100	-	-	-	-	-	-
Nanpin nongda 759	70S60	50MS60	60MR50	30R20	-	-	-	-	-	-
Pubingzi 4696	40MR30	30R20	40MR20	20R20	-	-	-	-	-	+
Nanpin yunong 82	40MR50	10MR10	60MR50	30MR20	-	-	-	+	+	-
Dehongfu 22	50MR40	0	70MR100	50MR100	-	-	-	+	+	-
Longxing 1	100S100	100S100	100S100	100S100	-	-	-	-	-	-
Nanpin yongfeng 308	100S100	100S100	100S100	60MS70	-	-	-	-	-	-
Yunong 937	100S100	100S100	100S100	20MS20	-	-	-	-	-	-
Huaguan 301	70MS50	100S100	100S100	70S80	-	-	-	-	-	-
Pingan25	20MR30	20MR20	30MR20	20MR20	-	-	-	+	+	-
Yutong 120	50MR60	20MR40	50MS50	30MS60	-	-	-	-	-	-
Nanpin M151	20MR10	5R5	30MR40	10R20	-	-	-	+	+	-
Luomai 66	100S100	40MS70	30MR40	20R20	-	-	-	-	-	-
Xinmai 85	30R60	20R30	10R10	5R10	-	-	-	+	+	-
Nanpin yongminmai 2	30R20	10R10	5R5	0	-	-	-	+	+	+
Xiezuo 1	100S100	60MS100	70S70	50S50	-	-	-	-	-	-
Jvliang 1208	0	0	20MR20	0	-	-	-	+	+	+
Zhengmai 916	0	0	30MR30	20R40	-	-	-	-	-	+
Xuke 16	50MR60	20MR20	10R20	5R10	-	-	-	+	+	-
Xinong 1152	20MR70	20R10	10R10	5R5	-	-	-	+	+	-
Xinong 635	40MR30	20MR20	80S80	50S50	-	-	-	-	-	-
Xinong 615	100S100	100S100	20MR10	30MR20	-	-	-	-	-	-
Zhengmai 216	100S100	100S100	30MS40	20MS40	-	-	-	-	-	-
Fumai 505	100S100	30S50	30MS60	10MS20	-	-	-	-	-	-
Xinfeng 88	20MR10	0	70MR80	50MR70	-	-	-	-	-	+
HG 2101	60MR50	5R5	30R20	5R5	-	-	-	+	+	-
Pumai 196	10R10	5R5	10R5	0	-	-	-	+	+	-
Yimai 211	0	0	0	0	-	-	-	-	-	+
Zhengmai 6166	10R10	0	5R5	0	-	-	-	-	-	-
Shunmai 17	50MR60	40MR30	20MR20	10MR10	-	-	-	+	+	-
Xuyan 6	10R10	0	10R20	5R5	-	-	-	+	+	-
Lunxuan 2116	70S70	50MS90	50MS40	60MS70	-	-	-	-	-	-
Puxing 35	60MS70	50MS60	50MS50	30MS40	-	-	-	-	-	-
Zhongyu 130	70MS80	60MS90	50MS50	40MS40	-	-	-	-	-	-
Shengmaiyuan 928	100S100	70S60	80S50	60MS70	-	-	-	-	-	-
Zhengxuan 219	30MR30	10R10	30MR50	5R5	-	-	-	+	+	-
gx2021	60MS50	5MS50	0	0	-	-	-	+	+	-
Luofeng 2101	20MR30	5R50	70S80	50S100	-	-	-	-	-	-
Tianyike 15	100S100	100S100	70S80	40MS40	-	-	-	-	-	-
Wanke 800	100S100	100S100	100S100	60S70	-	-	-	-	-	-
Huaimai 16659	70MS30	20R10	10R10	0	-	-	-	-	-	-
Baofeng 225	40MR40	5R5	50MR100	20R20	-	-	-	+	+	-
Yannong 5066	70MR70	20R10	20MR20	5R5	-	-	-	-	-	-
Bainongchengzu 21	10R10	0	0	0	-	-	-	+	+	-
Bainonghuarun 175	50MR50	10R10	10R10	10R10	-	-	-	+	+	-
Xinong 1522	10R10	0	10R10	0	-	-	-	-	-	-
Shannong 1695	20MR40	5R5	10R10	0	-	-	-	+	+	-
Shaanhe 285	100S100	100S100	100S100	50S50	-	-	-	-	-	-
XM 1186	70S70	30MS40	100S100	20MS50	-	-	-	-	-	-
Daimai 519	10R10	10R10	5R5	10R10	-	-	-	-	-	-
Xinong 816	0	0	0	0	-	-	-	+	+	-
Xinliang 18	20R40	20R10	20R50	40R20	-	-	-	+	+	-
LS01897	60MR70	40MR30	10R10	50R10	-	-	-	+	+	-
Xumai 19094	40MR50	10R10	5R5	5R5	-	-	-	+	+	-
Fumai 1699	50MR50	20R10	10R10	30R10	-	-	-	+	+	-
Yunong 612	100S100	100S100	100S100	100S100	-	-	-	-	-	-
Pubing 56	20MR30	0	5R5	0	-	-	-	+	+	-
Pubing 300	70MS80	50MS50	5R5	20R10	-	-	-	-	-	-
Zhongmai 236	80S100	50MS100	10R10	0	-	-	-	-	-	-
Xinong 5189	20MR40	10R20	20R30	20R10	-	-	-	+	+	-
Xinong 5199	30MR40	30MR40	0	10R10	-	-	-	+	+	-
Tianyike 18	20R50	10R10	5R5	5R5	-	-	-	+	+	-
Little Club	100S100	100S100	100S100	100S100	-	-	-	-	-	-

^a^ Infection responses: severity/infection type/incidence; severity and incidence rates in the table are percentages; R: resistant, MR: moderately resistant, MS: moderately susceptible, S: susceptible, 0: immune. ^b^ “+” indicates the presence of detected genes, while “-” indicates the absence of detected genes.

**Table 2:** Assessment of the resistance of tested wheat accessions to two races of *Pgt* at the adult plant stage.

Races	Adult Plant Stage					
	Immune		Resistant–Moderately Resistant		Moderately Susceptible–Susceptible	
	2023	2024	2023	2024	2023	2024
21C3CTHQM	5 (7.8) ^a^	12 (18.8)	35 (54.7)	29 (45.3)	24 (37.5)	23 (35.9)
34MKGQM	5 (7.8)	14 (21.9)	39 (60.9)	30 (46.9)	20 (31.2)	20 (31.2)
All races	2 (3.1)	8 (12.5)	35 (54.7)	30 (46.9)	27 (42.2)	26 (40.6)

^a^ 5 (7.8): The number outside the parentheses represents the number of cultivars, and the number inside the parentheses represents the percentage of that number.

**Table 3:** Effective/ineffective *Sr* genes against races 21C3CTHQM and 34MKGQM.

Race	Effective *Sr* Genes	Ineffective *Sr* Genes
21C3CTHQM	*5*, *9e*, *14*, *19*, *21*, *23*, *25*, *26*, *28*, *29*, *30*, *31*, *33*, *35*, *36*, *37*, *38*, *47*, *Tt3*	*6*, *7b*, *8a*, *9a*, *9b*, *9d*, *9f*, *9g*, *10*, *11*, *12*, *13*, *15*, *16*, *17*, *18*, *20*, *24*, *27*, *32*, *34*, *39*, *Tmp*, *GT*
34MKGQM	*9e*, *10*, *11*, *13*, *14*, *17*, *18*, *19*, *20*, *21*, *23*, *25*, *26*, *30*, *31*, *32*, *33*, *34*, *35*, *36*, *37*, *38*, *47*, *Tmp*, *Tt3*	*5*, *6*, *7b*, *8a*, *9a*, *9b*, *9d*, *9f*, *9g*, *12*, *15*, *16*, *22*, *24*, *27*, *28*, *29*, *39*, *GT*

**Table 4:** Host response and infection type descriptions used in wheat stem rust systems.

Host Response (Class)	Infection Type	Disease Symptoms
Immune	0	No uredinia or other macroscopic signs of infection
Very resistant	1	Small uredinia surrounded by necrotic tissue
Moderately resistant	2	Small to medium uredinia often surrounded by chlorosis or necrotic tissue; green islands may be surrounded by chlorosis or necrotic borders
Moderately susceptible	3	Medium-sized uredinia that may be associated with chlorosis
Susceptible	4	Large uredinia without chlorosis

**Table 5:** Primers linked to rust resistance genes and PCR conditions.

*Sr* Genes	Primer Namer	PCR Amplification Conditions	
		Temperature (°C)/Time	Number of Cycles
*Sr24*	Sr24#50	94/3 min	1
		94/30 s; 57/30 s; 72/40 s	30
		20/1 min	1
*Sr25*	Gb	94/3 min	1
		94/30 s; 60/30 s; 72/40 s	30
		20/1 min	1
*Sr26*	Sr26#43	94/3 min	1
		94/30 s; 56/30 s; 72/40 s	30
		20/1 min	1
*Sr31*	SCSS30.2_576_	95/5 min	1
		95/1 min; 60/1 min; 72/30 s	35
		72/10 min	1
*Sr31*	Iag95	94/3 min	1
		94/30 s; 55/60 s; 72/70 s	30
		25/60 s	1
*Sr38*	VENTRIUP-LN2	94/45 s	1
		94/45 s; 65/30 s; 72/1 min	30
		72/7 min	1

## Data Availability

Data are contained within the article.

## Supplementary Materials

The following supporting information can be downloaded at: https://www.mdpi.com/article/10.3390/plants13162286/s1, Table S1: Weather conditions in Shenyang from 26 May to 26 June, 2023 and 2024.
