# Supplementary material for: Stem Rust Resistance and Resistance-Associated Genes in 64 Wheat Cultivars from Southern Huanghuai, China
Source: Plants (Basel). 2024 Aug 17;13(16):2286. doi: 10.3390/plants13162286 (PMC11359684; doi:10.3390/plants13162286)
Supplement: Supplementary file 1 [file plants-13-02286-s001.zip › plants-3110122-supplementary.pdf]

Supplemental Table 1 Weather conditions in Shenyang from May 26th to June 26th, 2023 and 2024

Date		High temperature/ (℃)		Low temperature / (℃)		Weather		Wind direction	
2023	2024	2023	2024	2023	2024	2023	2024	2023	2024
5.26	5.26	30	20	15	12	Clear	Thunderstorm	South wind force 3	Northwest wind force 3
5.27	5.27	25	24	13	11	Cloudy	Clear	South wind force 3	North wind force 3
5.28	5.28	27	29	16	15	Clear	Clear	North wind force 1	Southwest wind 1
5.29	5.29	28	27	13	18	Clear	Cloudy	Northeast wind force 3	South wind force 3
5.30	5.30	27	26	14	13	Clear	Thunderstorm	Northeast wind force 3	Southwest wind force 3
5.31	5.31	28	21	17	11	Cloudy	Cloudy	South wind force 3	North wind force 1
6.1	6.1	27	25	13	12	Cloudy	Cloudy	Southwest wind force 3	Northwest wind force 1
6.2	6.2	27	25	12	10	Clear	Cloudy	North wind force 3	Northeast wind force 1
6.3	6.3	28	28	14	15	Clear	Clear	Northwest wind force 3	North wind force 2
6.4	6.4	30	30	17	17	Clear	Clear	Northwest wind force 3	South wind force 2
6.5	6.5	29	30	17	16	Clear	Cloudy	West wind force 3	Southwest wind force 3
6.6	6.6	29	29	19	18	Clear	Cloudy	Southwest wind force 3	Southwest wind force 3
6.7	6.7	28	26	14	17	Thunderstorm	Thunderstorm	Southwest wind force 3	South wind force 3
6.8	6.8	27	27	13	17	Cloudy	Light rain to moderate rain	Southwest wind force 3	South wind force 1
6.9	6.9	23	28	14	15	Thunderstorm	Clear	South wind force 3	Northwest wind force 1
6.10	6.10	24	33	14	20	Thunderstorm	Clear	South wind force 3	Southwest wind force 3
6.11	6.11	24	32	14	19	Thunderstorm	Thunderstorm	Northwest wind force 3	South wind force 3
6.12	6.12	24	31	12	20	thunderstorm	Clear	Southwest wind force 3	South wind force 1

6.13	6.13	26	31	13	19	Clear	Thunderstorm	Northeast wind force 1	Southwest wind force 3
6.14	6.14	29	26	16	17	Clear	Thunderstorm	South wind force 1	Southwest wind force 1
6.15	6.15	31	26	19	15	Clear	Clear	Southwest wind force 3	North wind force 1
6.16	6.16	34	31	19	21	Clear	Clear	Southwest wind force 3	Southwest wind force 1
6.17	6.17	35	35	17	21	Clear	Clear	Southwest wind force 3	North wind force 1
6.18	6.18	33	35	18	23	Clear	Clear	South wind force 1	Southwest wind force 1
6.19	6.19	34	35	20	20	Cloudy	Clear	South wind force 3	Southwest wind force 3
6.20	6.20	24	32	18	18	Thunderstorm	Cloudy	South wind force 2	Southwest wind force 3
6.21	6.21	25	30	17	21	Cloudy	Cloudy	North wind force 3	Southwest wind force 1
6.22	6.22	30	28	19	18	Clear	Light rain to moderate rain	North wind force 3	Southwest wind force 1
6.23	6.23	33	23	21	15	Clear	Cloudy	South wind force 3	North wind force 1
6.24	6.24	35	24	19	14	Clear	Cloudy	South wind force 3	North wind force 3
6.25	6.25	34	28	20	18	Clear	Clear	South wind force 3	South wind force 1
6.26	6.26	30	32	20	19	Thunderstorm	Clear	South wind force 3	South wind force 1
